# Myeloma-specific superenhancers affect genes of biological and clinical relevance in myeloma

**DOI:** 10.1038/s41408-021-00421-7

**Published:** 2021-02-12

**Authors:** Yunlu Jia, Jianbiao Zhou, Tze King Tan, Tae-Hoon Chung, Regina Wan Ju Wong, Jing-Yuan Chooi, Julia Sze Lynn Lim, Takaomi Sanda, Melissa Ooi, Sanjay De Mel, Cinnie Soekojo, Yongxia Chen, Enfan Zhang, Zhen Cai, Peng Shen, Jian Ruan, Wee-Joo Chng

**Affiliations:** 1 Cancer Science Institute of Singapore, National University of Singapore, 14 Medical Drive, Centre for Translational Medicine, Singapore, 117599 Republic of Singapore; 2 Department of Medical oncology, the First Affiliated Hospital, Zhejiang University School of Medicine, Hangzhou, China; 3 Department of Medicine, Yong Loo Lin School of Medicine, National University of Singapore, Singapore, 117597 Republic of Singapore; 4 Department of Hematology-Oncology, National University Cancer Institute of Singapore (NCIS), The National University Health System (NUHS), 1E, Kent Ridge Road, Singapore, 119228 Republic of Singapore; 5 Department of Surgical Oncology, Sir Run Run Shaw Hospital, College of Medicine, Zhejiang University, Hangzhou, China; 6 Bone Marrow Transplantation Center, the First Affiliated Hospital, Zhejiang University School of Medicine, Hangzhou, 310003 China

**Keywords:** Cancer, Cell biology

## Abstract

Multiple myeloma (MM) is an aggressive plasma cell neoplasm characterized by genomic heterogeneity. Superenhancers (SEs) are defined as large clusters of enhancers in close genomic proximity, which regulate genes for maintaining cellular identity and promote oncogenic transcription to which cancer cells highly addicted. Here, we analyzed *cis*-regulatory elements in MM samples with H3K27ac ChIP-seq, to identify novel SE-associated genes involved in the myeloma pathogenesis. SEs and their associated genes in cancerous tissue were compared with the control samples, and we found SE analysis alone uncovered cell-lineage-specific transcription factors and well-known oncogenes ST3GAL6 and ADM. Using a transcriptional CDK7 inhibitor, THZ1, coupled with H3K27ac ChlP-seq, we identified MAGI2 as a novel SE-associated gene of myeloma cells. Elevated MAGI2 was related to myelomagenesis with gradual increased expression from MGUS, SMM to newly diagnosed and relapsed MM. High prevalence of MAGI2 was also associated with poor survival of MM patients. Importantly, inhibition of the SE activity associated with MAGI2 decreased MAGI2 expression, inhibited cell growth and induced cell apoptosis. Mechanistically, we revealed that the oncogenic transcription factor, MAF, directly bound to the SE region and activated gene transcription. In summary, the discoveries of these acquired SEs-associated genes and the novel mechanism by which they are regulated provide new insights into MM biology and MAGI2-MAF-SE regulatory circuit offer potential novel targets for disease treatment.

## Introduction

Multiple myeloma (MM) is characterized by neoplastic proliferation of plasma cells in the bone marrow, which originate from the post-germinal lymphoid B-cell lineage^1^. Due to the genetic and epigenetic heterogeneity, MM remains an almost incurable disease^2,3^. Worldwide, MM is the second most common blood cancer and results in over 100,000 deaths per year^4^. The dissection of the molecular landscape of MM would lead to a better understanding of the disease biology and the development of more effective therapeutic options.

Enhancers are distal DNA regulatory elements and central regulators of precisely gene expression programs^5^. Accumulating evidence has demonstrated that transcriptions of key oncogenes and maintenance of cancer cell identity are driven by large clusters of enhancers, called superenhancers (SEs)^6,7^. In general, SE elements are highly occupied by active enhancer marks (H3K27ac, H3K4me1), mediator complex (MED), P300, bromodomain-containing protein 4 (BRD4), cyclin-dependent kinase 7 (CDK7), and master transcription factors (TFs). Previous studies revealed that SEs facilitated high-level transcription of key regulators in MM cell state, such as *IGLL5*, *MYC*, *IRF4*, and *PRDM1/BLIMP-1*^8,9^. A recent combinatorial analysis of gene expression, open chromatin, and enhancer landscape further revealed the aberrant TF regulatory network and the epigenetic changes of MM^10^. However, because MM consists of several molecular genetic subgroups, the identification of specific SE gene and its regulatory elements in certain subsets of patients has remained unexplored. Such information will be useful for the development of precision therapeutics for a disease with heterogeneously genetic background, like MM.

Considering the transcriptionally dependence of these functionally relevant SE-driven genes, the expression of these genes would be significantly repressed upon transcriptional inhibition. JQ1 (a competitive inhibitor of BRD4) and THZ1 (a selective CDK7 inhibitor) target different critical components of SEs, which are effective transcriptional inhibitors that have shown anti-tumoral effects against various cancers^7,11^. Previously, using a strategy of first identifying SE-associated genes by H3K27ac ChlP-Seq followed by further filtering those SE-associated genes whose expression are significantly downregulated following THZ1 treatment, we have identified functionally relevant SE-associated genes in T-ALL^12^.

In the current study, we employed a similar strategy to identify functionally relevant SE-associated genes of myeloma cells. In particular, we search for MM-acquired SE-genes that are especially associated with unique genetic subtypes of myeloma and further examine their role in disease pathogenesis.

## Results

### Characterization of SEs landscape of representative MM samples and cell lines

**Fig. 1:**
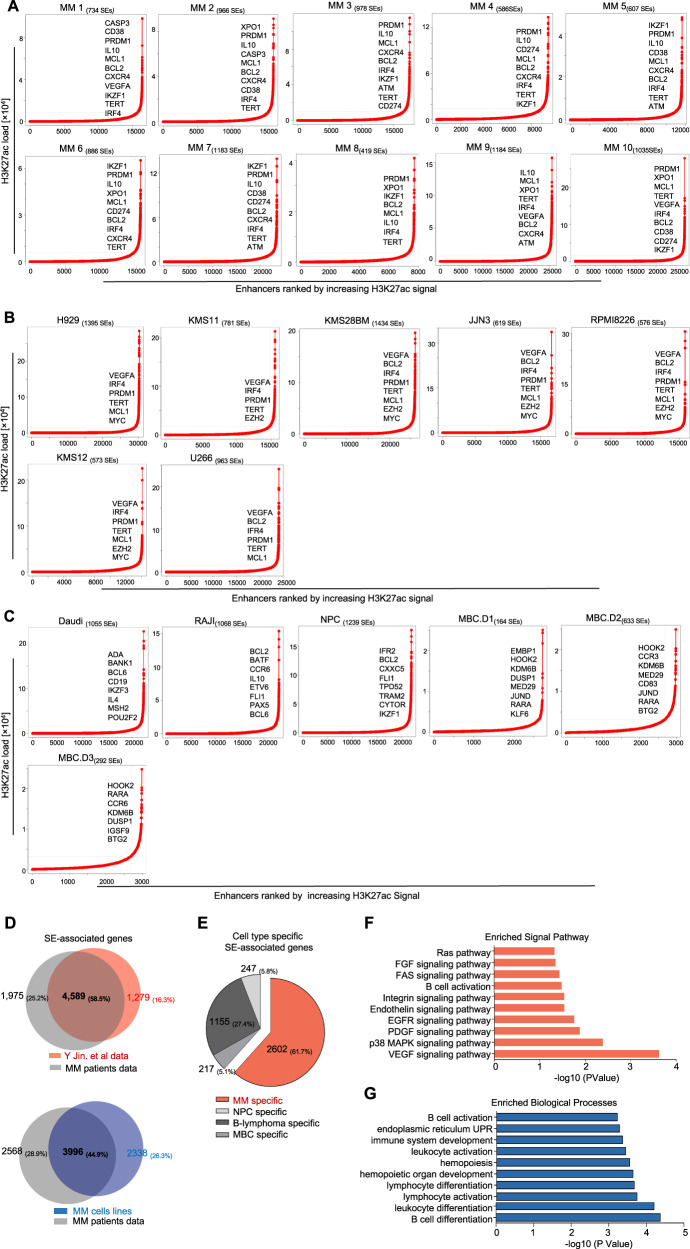
SEs landscape of patients-derived MM samples and MM cell lines. **A–C** Enhancer regions of ten primary MM cases (MM#1–10) (**A**), MM cell lines (**B**) and the control cells (including normal plasma cell (NPC), B-lymphoma cell lines (RAJI and Daudi), three memory B cells (MBC.D1, MBC.D2, and MBC.D3) (**C**). Enhancers were ranked by increasing H3K27ac signal, and enhancers above the inflection point of the curve were defined as SEs. The number of SEs was shown for each sample, and examples of SEs-associated genes found in at least five primary MM cases were also presented. **D** SE-associated genes of in-house primary MM samples were compared with those collected from another independent MM-SE-genes dataset^10^ (top) and MM cell lines (bottom). **E** Cell type specific SE-associated genes were presented in MM (2602), NPC (247), B-lymphoma cells (1155), and MBC (217). **F** MM-specific SE-genes were enriched in multiple cancer-relating signaling pathways, such as Ras and EGFR pathway. **G** Gene Ontology (GO) molecular functions of MM-specific SE-associated genes were associated with biological processes essential to the biology of MM.

To investigate the SE profiles in MM, H3K27ac chromatin immunoprecipitation followed by massively parallel DNA sequencing (ChIP-seq) was performed on selected untreated patient-derived MM samples (MM 1–10) and HMCLs (H929, KMS11, KMS28BM, JJN3, RPMI8226, KMS12, and U266) with different known translocations. List of HMCLs and baseline clinicopathological characteristics and genetic information of patient-derived MM samples included in this study were presented in Supplemental Table 1 and Table 2, respectively. As for the controls, we selected one normal plasma cell (NPC), two B-lymphoma cell lines (Daudi and RAJI), and three memory B cells (MBC.D1, MBC.D2, and MBC.D3)^10^. We also analyzed publicly-available H3K27ac ChIP-seq datasets containing additional MM patient cases (MM 11–20) and HMCLs (MOLP-2, LP-1, MM1.S, and OPM2)^10^. Using these datasets, SEs and SEs-associated genes were identified in each of samples (Fig. 1A–C and Supplementary Fig. 1A). Several SE-associated genes presented in at least five MM samples were indicated beside the SE curves. These commonly identified SE-genes included *BCL2, IRF4*, *MCL1, CD38*, and *IL10*. These SE-genes are frequently overexpressed in human MM and essential for the survival of MM cells and maintenance of cancer cells state^13–15^, thus consistent with findings by other groups. The complete list of the SE-genes was presented in Supplemental Table 3–5.

Next, we compared SE-associated transcripts from two independent datasets of primary MM samples, HMCLs with the controls. A total of 4589 SE-genes (58.5%) were commonly found in the public dataset for MM samples and our samples. We also observed 44.9% of the SE-genes were shared between primary MM samples and HMCLs (Fig. 1D). These shared SE-genes among different MM samples are valuable for further study. When compared with the control cells, a total of 2602 MM-acquired SE-associated genes (MM-SE genes) were identified after excluding 247 SE-genes unique to NPC, 1155 SE-genes unique to B-lymphoma cells, and 217 SE-genes unique to MBC (Fig. 1E). Signaling pathway analysis revealed that these MM-SE genes were highly enriched in key pathways related to cancer development and progression, including Ras, p38 MAPK, and EGFR signaling pathway (Fig. 1F). These MM-SE genes were also highly enriched in essential biological processes during MM pathogenesis, including B-cell differentiation, cellular response to unfolded protein, and immune system development. Moreover, genes involved in cancer-related functions such as cell proliferation and apoptosis were also significantly overrepresented in MM-SE-genes (Fig. 1G).

### SEs were enriched in known oncogenes essential for MM pathogenesis

**Fig. 2:**
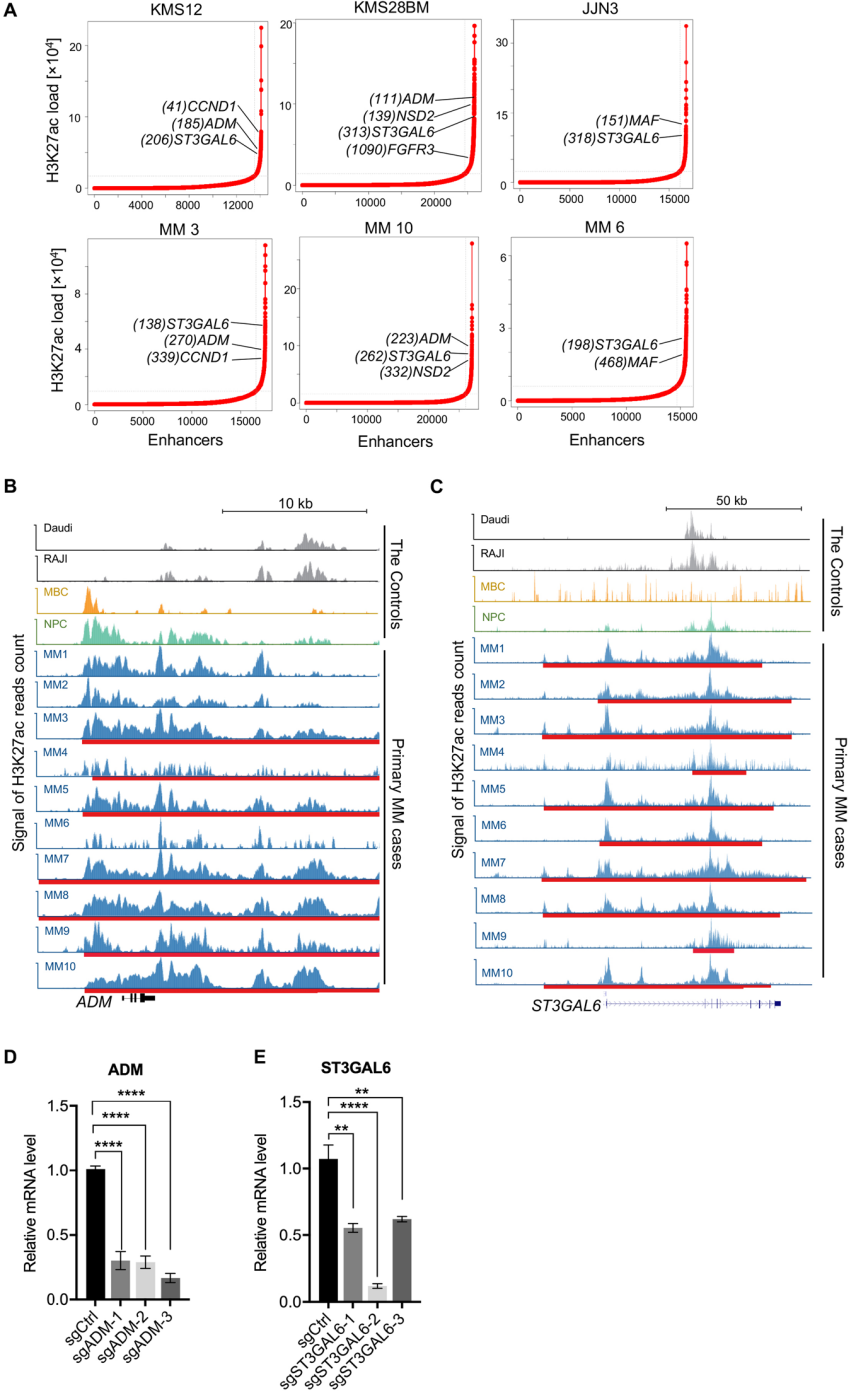
SEs were enriched at various well-known oncogenes in MM. **A** Enhancers in MM cell lines (KMS12, KMS28BM, and JJN3) and patient-derived myeloma tissue (MM3, MM6, and MM10) were ranked by average H3K27ac signal. SEs were determined by the inflection point of the plot and SEs-associated genes were annotated along the vertical axis and. **B, C** Gene tracks of H3K27ac ChIP-seq signal across patient-derived myeloma tissue (MM1-MM10) and the control samples at the *ST3GAL6* (**B**) and *ADM* (**C**) locus. The amplicon and SEs were shown (red line). **D, E** mRNA expression of *ST3GAL6* and *ADM* genes in H929 cell upon CRISPR-mediated repression of the indicated SE. H929 cell coexpressing dCas9 was infected with target-specific sgRNAs at various positions within the indicated *ADM*-SE region (sgADM-1, sgADM-2, sgADM-3) (**D**), *ST3GAL6*-SE region (sgST3GAL6-1, sgST3GAL6-2, sgST3GAL6-3) (**E**) or control sgRNAs (sgCtrl). mRNA expression relative to nontransduced cells is shown as mean ± SEM. *P < 0.05, **P < 0.01, ***P < 0.001, ****P < 0.0001 by two-sample, two-tailed *t*-test compared with the control. Experiments were performed in triplicate.

Next, we analyzed the H3K27ac signals and found SEs were associated with oncogenic drivers previously shown to be essential for MM pathogenesis. When ranked against H3K27ac signal across all enhancer regions, the β-galactoside α-2,3-sialyltransferase (*ST3GAL6)* and adrenomedullin (*ADM)* were among these top-ranked SE-associated genes. Interestingly, MM samples with t(11;14), t(4;14), and t(14;16) translocations displayed de novo SE formation at translocated *CCND1*, *FGFR3/MMSET*, and *MAF* loci, respectively, potentially formed as a consequence of altered chromatin topology (Fig. 2A). Previously *ST3GAL6* was implicated as an oncogenic driver in MM, and *ST3GAL6* silencing inhibited cell motility of back to the bone marrow niche^16^. Similarly, aberrantly expressed *ADM* was a critical driving force for the angiogenic switch during MM progression^17^. Here, we observed significant H3K27ac signals enriched near *ST3GAL6* and *ADM* genes loci across most MM patients’ samples and HMCLs. In contrast, only background level signals were present in the control samples (Fig. 2B–C). Indeed, SE formation observed at these two genes loci were unique to MM cases, which may lead to aberrant overexpression of *ST3GAL6* and *ADM*, compared with bone marrow plasma cells (BMPC) and non-MM hematological malignancies (data collected from Amazonia, http://amazonia.transcriptome.eu/, Supplementary Fig. 2A–B). Furthermore, we confirmed that CRISPR-dCas9-mediated inhibition of the SE region caused decreased *ST3GAL6* and *ADM* expression, respectively (Fig. 2D–E). Consistently with previous studies, higher expression of *ST3GAL6* or *ADM* was correlated with faster MM disease progression and unfavorable outcome (Supplementary Fig. 2C–D). These observations suggested that SEs were involved in driving expression of clinically and biological relevant genes in MM pathogenesis.

MM patients carrying t(14;16) was associated with poor survival and did not benefit from the addition of bortezomib. Early studies revealed that high levels of *MAF* due to the translocation t(14;16)(q32;q23) was seen in 25% of HMCLs, and increased *MAF* expression was also detected in several t(4;14)-positive myeloma cells with uncertain mechanisms^18^. Here, we found the formation of a novel SE at *MAF* gene loci in myeloma cells carrying t(14;16) (MM6, JJN3, RPMI8226, and KMS11) and H929 cell, one t(4;14)-positive cell with aberrant *MAF* protein activation (Supplementary Fig. 2E). The strong activation of *MAF* here could be the result of this newly MM-acquired SE. Similarly, *NUAK1*, a molecular determinant of malignant MM, is transcriptionally regulated by the Large-MAF family^19^. We identified the formation of de novo SE at the *NUAK1* loci, which was unique to the t(14;16)-positive cases, including MM6, JJN3, and MM1.S (Supplementary Fig. 2F). Taken together, these results demonstrated that the strong activation of general oncogenes, as well as several genetic subgroup-specific oncogenic drivers, was the results of active SEs formation in MM.

### THZ1 inhibited RNA polymerase II-mediated transcription of myeloma cells

**Fig. 3:**
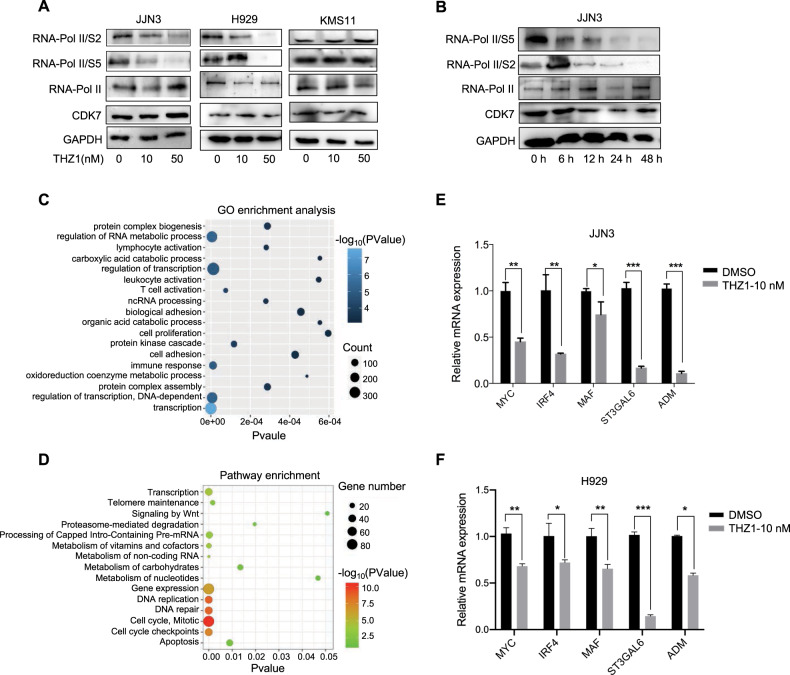
SEs-associated genes were sensitive to CDK7 inhibition. **A** Immunoblotting analysis of RNA polymerase II (RNAPII) C-terminal domain (CTD) phosphorylation in two THZ1-sensitive (JJN3 and H929) and one THZ1-resistant (KMS11) cell line, which were treated with either THZ1 (10 nM, 50 nM) or DMSO for 24 h. **B** Treatment of THZ1 (50 nM) diminished the phosphorylation of serine 2 and 5 on Pol II CTD of JJN3 in time-dependent manner. **C, D** Gene ontology enrichment analysis and pathway analysis showed that THZ1-sensitive genes were related to transcription regulation, DNA repair and apoptosis regulation. **E, F** JJN3 and H929 cells treated with THZ1 or vehicle were analyzed by qRT-PCR for SE-genes *MYC, IRF4, MAF, ST3GAL6*, and *ADM* expression, normalized against GAPDH. Each bar represents the mean ± SEM of three independent experiments. *p < 0.05, **p < 0.01, ***p < 0.001, ****p < 0.0001 by two-sample, two-tailed *t*-test compared with the DMSO-treated control.

Transcriptional dependencies that are sustained by SEs tend to be selectively targeted by CDK7 inhibition in various cancer types^8,20^. THZ1 is a novel covalent inhibitor of CDK7, which has been shown to be highly effective in killing tumor cells^8,20–23^. Here we tested the sensitivity of various HMCLs to THZ1. Generally, HMCLs were sensitive to CDK7 inhibition, with IC50 values ranging from 10 to 450 nM. KMS11 cell presented intrinsic resistant to THZ1, while JJN3 and H929 were two relatively THZ1-sensitive cells (Supplementary Fig. 3A, B). Consistent with THZ1’s known effect on transcription by inhibiting CDK7, THZ1 treatment diminished the phosphorylation of Serine 2 and 5 on Pol II CTD in the two sensitive cell lines (JJN3 and H929) in a dose-and time-dependent manner, but not in the resistant cell (KMS11) (Fig. 3A, B). To further understand the transcriptional vulnerability to THZ1 in HMCLs, we performed genome-wide expression profiling before and after THZ1 treatment in JJN3 and H929 cells (50 nM, 24 h), followed by gene ontology (GO) enrichment analysis and pathway analysis. Notably, transcription process and regulation of transcription were among the most significant gene ontology categories suppressed by THZ1 in myeloma cells, further indicating THZ1’s inhibitory effect on CDK7-dependent production of transcriptional regulators (Fig. 3C–D).

In general, SE-associated genes were more sensitive to THZ1 as compared with those genes regulated by typical enhancers (TEs). We found that mRNA expression levels of previously-reported oncogenic TFs, such as *MAF*, *MYC*, *IRF4*, and MM-specific SE-associated oncogenes *ST3GAL6* and *ADM* were significantly downregulated upon THZ1 treatment (10 nM, 24 h) (Fig. 3E–F). Notably, THZ1 has no or limited effect on the expression of housekeeping genes such as GAPDH and TUBA1A, which are regulated by TEs (Supplementary Fig. 3C). These results indicated the selective effects of CDK7 inhibition on SE-associated genes in myeloma, which was consistent with previous studies in other cancer types. We further proposed that THZ1-sensitive transcripts amongst the SE-associated genes are likely to be the more functionally relevant genes.

### Identification SEs-associated oncogenes of clinical relevance

**Fig. 4:**
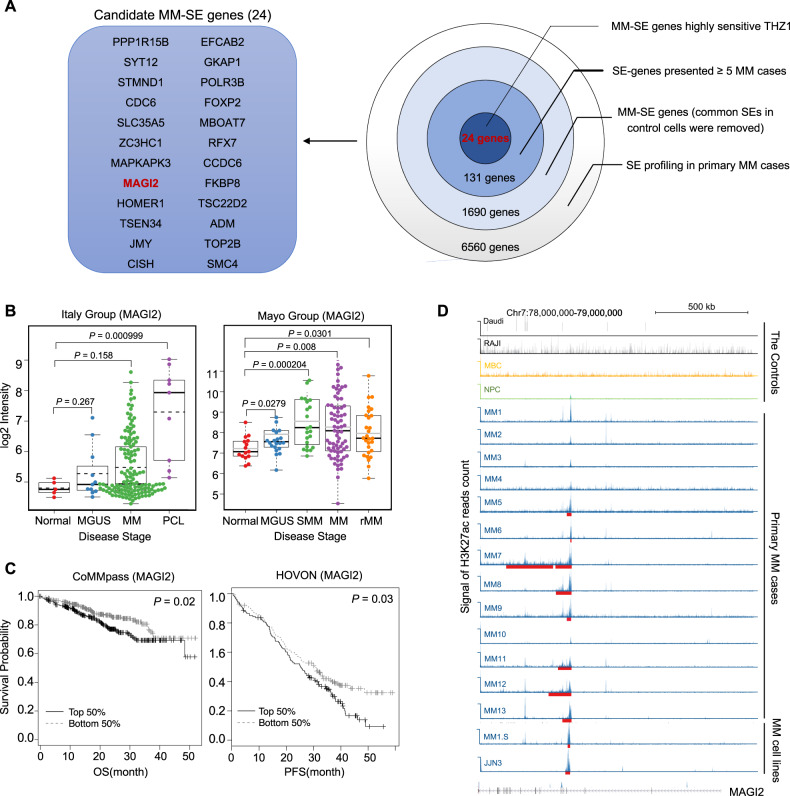
Selection of SE-associated oncogenes with clinical significance. **A** Schematic of the selection strategy, and 24 candidate MM-SE genes were selected from this analysis. **B** Based on Mayo and Italy myeloma datasets, MM-SE gene *MAGI2* contributed to myelomagenesis with gradual increasing expression from MGUS, SMM to newly diagnosed and relapsed MM or plasma cell leukemia. **C** Kaplan–Meier survival curves showed higher *MAGI2* expression level was associated with poor survival of MM patients. Significance was evaluated by Log-rank test. **D** The ChIP-seq gene tracks represented strong H3K27ac signals within *MAGI2* gene loci in patient-derived MM samples and MM cell lines (JJN3 and MM1.S), but not presented in the control cases (NPC, MBC, RAJI, and Daudi). The amplicon and SEs were shown (red line).

Considering functionally relevant SE-associated oncogenes would be strongly expressed in MM tumors and highly dependent on continuously active transcription, we searched for candidate SE-associated oncogenes using the following criteria: (1) MM-SE genes presented in at least five primary MM cases, but not shared in control samples, and (2) MM-SE genes sensitive to THZ1 treatment (adj.p value < 0.05, Log2 (FC) > 0.5). Correspondingly, 24 candidate MM-SE genes were finally selected from this analysis (Fig. 4A). We validated that expression levels of these selected SE-genes were highly sensitive to transcription inhibition in JJN3 cell upon THZ1 treatment (50 nM, 24 h) (Supplementary Fig. 4A). In contrast, in the THZ1-resistant MM cell, KMS11, the selected SE-genes were either only modestly decreased or remained unaltered upon exposure to THZ1 (Supplementary Fig. 4B). Additionally, another SE blocker, BET inhibitor JQ1 (50 nM) significantly reduced most of the SE-genes transcription in JJN3 cell as demonstrated by qRT-PCR analysis (Supplementary Fig. 4C). Indeed, JQ1 causes preferential loss of BRD4, Mediator, and P-TEFb binding to SE region and decreased transcription of SE-associated genes^5^.

Integrated bioinformatics analysis with expression profile revealed several MM-SE genes were potential biomarkers in predicting clinical outcome and myeloma disease progression. From the Mayo Clinic and Italian Group MM datasets, six MM-SE genes (*SLC35A5, MAGI2, HOMER1, TSC22D2, ADM*, and *SMC4*) may contribute to MM disease progression (Supplementary Table 6). As an example, we presented that elevated *MAGI2* was related to faster MM disease progression (Fig. 4B). Among these selected MM-SE genes, Kaplan–Meier analysis was applied to compare overall survival (OS) and progression-free survival (PFS) based on gene expression levels. We identified that overexpression of *MAGI2* was significantly associated with unfavorable outcome in MM patients (Fig. 4C). *MAGI2* is a membrane-associated guanylate kinase family protein with multiple PDZ domains^24^. This gene has a very large intronic region, which function as scaffold proteins to assemble multiprotein signaling complexes^25^. In this analysis, significant H3K27ac signal at *MAGI2* introns and the coding region was found of in-house patient-derived MM tissues (MM 1–10), also in another independent MM dataset (MM 11–13), and HMCLs (JJN3 and MM1.S), but not in the control samples (Fig. 4D). Taken together, SEs and SEs-associated genes with clinical relevance in MM may be required for sustaining cancer development and act as promising therapeutic targets.

### *MAGI2* was a novel SE-associated oncogenic driver in MM

**Fig. 5:**
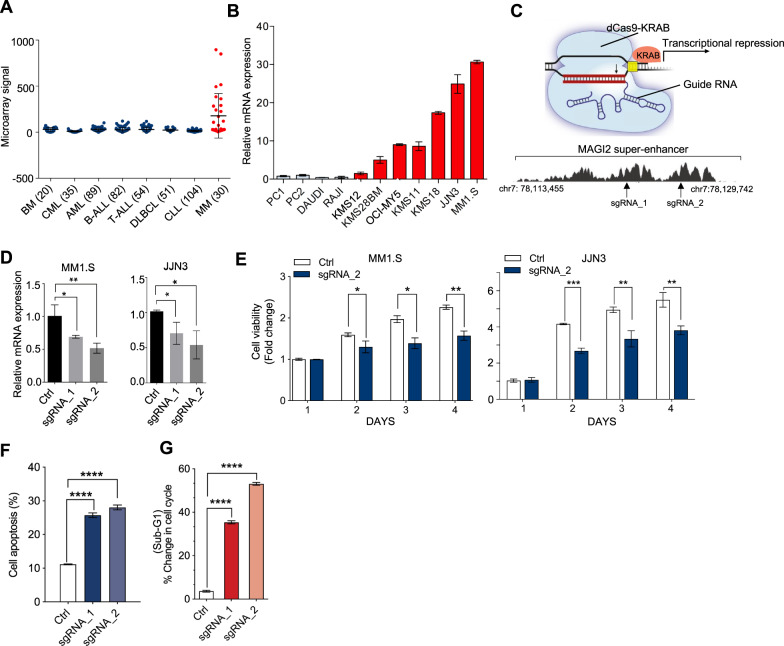
*MAGI2* was required for MM cell growth and proliferation. **A** *MAGI2* expression was significantly upregulated in MM samples when compared to non-MM hematological malignancies (CML, AML, B-ALL, T-ALL, DLBCL, and CLL). (Data collected from Amazonia, http://amazonia.transcriptome.eu/). **B** mRNA expression levels of *MAGI2* gene in various MM cell lines, NPC, RAJI, and Daudi were measured by qRT-PCR. **C** Upper panel: A schematic diagram of the sgRNA directed dCas9-KRAB transcription repression system; Lower panel: sgRNAs designed to target multiple sites of *MAGI2-*SE region. **D** Expression of *MAGI2* upon CRISPR/dCas9-mediated targeting repression of the indicated SE in JJN3 and MM1.S cells. The quantified *MAGI2* mRNA expression is shown. **E** Blockade of *MAGI2*-SE abrogated MM growth in vitro. Relative absorbance by cell viability assays (*y*-axis) at different days of culture (*x*-axis) is shown. **F** Cell deaths were determined on 72 h after *MAGI2-*SE inhibition in MM1.S cells, which were stained with annexin V and propidium iodide (PI) and analyzed by flow cytometer. **G** Deletion of *MAGI2*-SE in MM1.S caused an increased proportion of sub-G1 cells. *p < 0.05, **p < 0.01, ***p < 0.001, ****p < 0.0001 by two-sample, two-tailed *t*-test compared with the controls. Error bars indicate the mean ± SEM. Experiments were performed in triplicate.

Generally, oncogenic SEs promote tumorigenesis via activating oncogene transcription or mediating the dysregulation of signaling pathways. Here, we chose *MAGI2* gene for further study. As expected, *MAGI2* was expressed much higher in MM specimens than normal bone marrow tissues (BM) and other non-MM hematological malignancies (data collected from Amazonia, Fig. 5A). qRT-PCR assay also confirmed the elevated expression of *MAGI2* in a range of HMCLs compared with the normal plasma cells (PC1 and PC2), Daudi and RAJI (Fig. 5B). Notable, we observed MM1.S and JJN3 cells with identified new SE formation displayed the highest expression levels of *MAGI2*.

*MAGI2*-SE is located in the DNA region of chr7: 78103091–78128238, which is around 500 kb downstream from the TSS site. To assess the causal link between the SE and *MAGI2* dysregulation in MM, we used the CRISPR/dCas9 genome editing with guide RNA designed to target the SE sites (Fig. 5C). Repression of the SE within the *MAGI2* region significantly decreased *MAGI2* expression in both JJN3 and MM1.S cells (Fig. 5D). As expected, we observed H3K27ac, indicative of active enhancers, was dramatically lost at the SE region of *MAGI2* locus upon CRISPR/dCas9-KRAB mediated suppression compared to other tested conditions (Supplementary Fig. 5A). Cellular function assay revealed that suppression of the *MAGI2*-SE function caused the impairment of cell viability, and enhancement of cell apoptosis, consistent with an oncogenic function (Fig. 5E–G, Supplementary Fig. 5B–C). Overall, our results demonstrated that activation of SEs represent a common mechanism to activate novel cancer driver genes, and suggested a potential oncogenic role of *MAGI2* in myeloma progression.

### *MAF* bound to the SE region of *MAGI2* and transactivated gene transcription

**Fig. 6:**
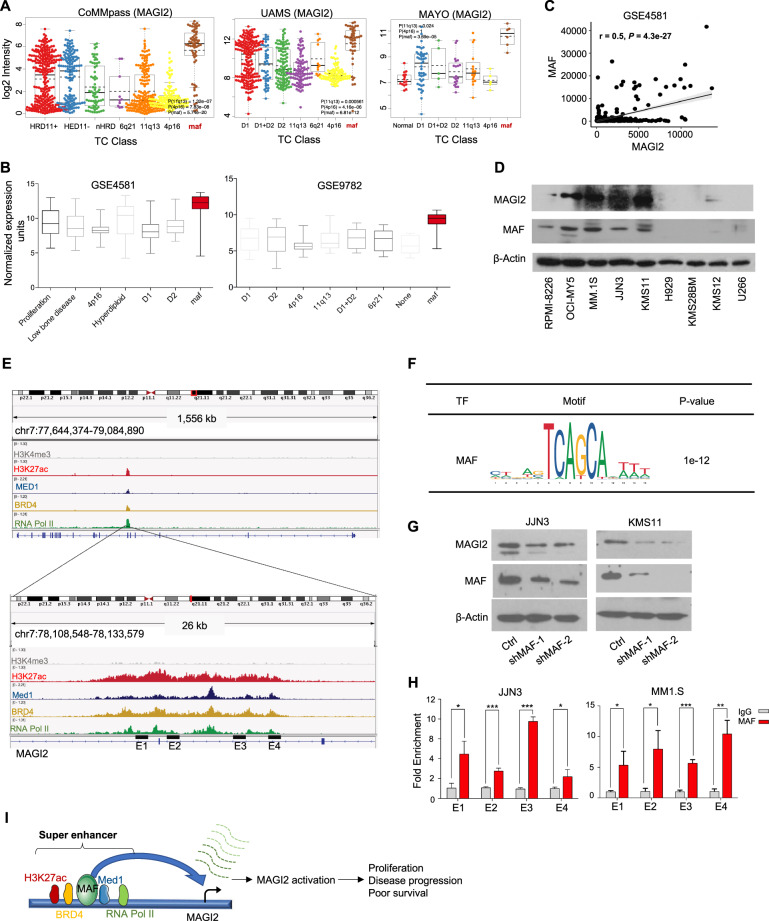
*MAF* directly bound to the super enhancer and activated *MAGI2* transcription. **A, B** *MAGI2* expression in different genetic subtypes of MM, in which its expression is the highest in the *MAF*-positive MM subgroup (data based on CoMMpass, UAMS, Mayo myeloma datasets, GSE4581 and GSE9782). **C** Correlation analyses were utilized to quantify the association between *MAGI2* and *MAF* expression levels. A potential positive correlation of *MAGI2* and *MAF* gene was found in clinical specimens of MM (GSE4581). **D** Global protein expression levels of *MAGI2* and *MAF* in a range of MM cell lines. Elevated *MAGI2* protein in MM cells was accompanied by high levels of *MAF*. **E, F** The ChIP-seq gene tracks represent H3K27ac, H3K4me3, MED1, BRD4, and RNA pol II signals at the *MAGI2* gene loci, and *MAF* binding site locates within the *MAGI2*-SE loci. **G** The protein levels of *MAGI2* were detected in JJN3 and KMS11 cells transfected with *MAF* shRNA or Ctrl by western blotting. The expression of β-actin was detected as a protein loading control**. H** ChIP-qPCR assays against *MAF* at the predicted genomic binding sites within *MAGI2*-SE region were presented in JJN3 and MM1.S cells. **I** Schematic diagram showing SE-driven *MAGI2* activation is dependent, at least partially, on oncogenic transcription factor *MAF* in MM. *p < 0.05, **p < 0.01, ***p < 0.001, ****p < 0.0001 by two-sample, two-tailed *t*-test compared with the control. Error bars indicate the mean ± SEM. Experiments were performed in triplicate.

When assessing the association of *MAGI2* expression with the genetic subtypes of MM, we noted that its appearance was the highest in the *MAF*-positive subgroup of several independent MM datasets (Fig. 6A–B, Supplementary Fig. 6A, B). Based on the expression profile, there was a modest positive correlation between *MAGI2* and *MAF* mRNA expression levels (Fig. 6C, Supplementary Fig. 6C). Western blot results also showed that increased protein level of *MAGI2* was detected in the HMCLs harboring overexpressed *MAF* protein (OCI-MY5, MM1.S, JJN3, and KMS11) (Fig. 6D). *MAF* is a key oncogenic transcription factor that overexpressed in more than 50% of MM and contributes to myelomagenesis. MM patients bearing *MAF* elevation are of poor prognosis and targeting *MAF* could be a therapeutic strategy^18,26,27^.

We further investigated if this positive correlation is due to *MAF* binding to the SE of *MAGI2* gene. Analyzing ChIP-seq data for motif discovery via HOCOMOCO, several binding sites of *MAF* were located within the *MAGI2*-SE region (Fig. 6E–F). *MAF* binding to the *MAGI2*-SE was also predicted with another online SE analysis tool^28^ (Supplementary Table 7). Next, we found that silencing of *MAF* in JJN3 and KMS11 cells caused a subsequently decreased *MAGI2* expression (Fig. 6G). Moreover, we performed ChIP-qPCR with anti-MAF, confirming that the ChIP enrichment signal of *MAF* was specific within SE region of *MAGI2* (Fig. 6H). Collusively, we presented the schematic diagram showing SE-driven *MAGI2* activation is dependent, at least partially, on oncogenic transcription factor *MAF* in MM (Fig. 6I).

Taken together, the SE region of *MAGI2* is bound by *MAF*, further providing a mechanism by which overexpressed transcription factor in driving expression of important downstream oncogenic genes by interacting with the SE.

## Materials and methods

### Cell culture and reagents

All human myeloma cell lines (HMCLs) used in this study were grown in RPMI-1640 medium (BioWest) containing 10% fetal bovine serum (FBS) and 1% penicillin-streptomycin and kept at 37 °C with 5% CO_2_. The identity of HMCLs was recently authenticated by short tandem repeat analysis. Plasma cells were isolated by CD138 immunomagnetic bead selection from bone marrow aspirates of newly diagnosed patients with MM, which were obtained from the National University Hospital after approval by the institutional ethical committee. Reagents and kits included: DMSO was purchased from Sigma. THZ1 (1604810-83-4) was obtained from Cayman Chemical. JQ1 (SML0974) was purchased from Sigma–Aldrich. Antibodies used are shown in Supplemental information.

### Cell viability assay

The Cell Titer-Glo Luminescent Cell Viability Assay Kit (Promega) was used and readings were recorded using the Tecan Infinite 200 PRO plate reader (Tecan). The IC_50_ values were calculated by nonlinear regression analysis using GraphPad Prism6.

### Identification and analysis of superenhancers

ChIP-seq was performed in patients-derived myeloma samples and HMCLs using polyclonal anti-H3K27ac (Abcam, ab4729). ChIP-seq datasets were aligned to the hg19 human genome by Bowtie 22.3.4.1. Regions of H3K27ac ChIP-seq peaks were identified by MACS2 1.4.2. Constituent enhancers that occurred within 12.5 kb were further stitched together and excluded those were fully contained within ±2kb from TSS for SE identification by ROSE2 with the parameter –s 12,500 and –t 2000. Enhancer regions were plotted in an increasing order based on their H3K27ac ChIP-Seq signal. Enhancers above the inflexion point of the curve were defined as SEs. SEs were assigned to genes with TSS flanking a 50 kb window of the SEs. Library construction and sequencing on the Illumina HiSeq 4000 platform were performed by BGI Tech Solutions Co., Limited (Hong Kong). All H3K27ac ChIP-Seq data were deposited in the GEO database (Accession number: GSE 145938)

### RNA-seq and data analysis

Total RNA was extracted using the RNeasy mini kit (Qiagen). RNA-Seq was performed in JJN3 and H929 cells treated with THZ1 (50 nM, 24 h) or with the vehicle control (DMSO). The RNA library construction and RNA-sequencing services were provided by BGI Tech Solutions Co., Limited (Hong Kong). RNA-seq reads were aligned to the hg19 human reference genome using STAR 2.5.2a with outFilterMultimapNmax set to 1. Total mapped reads were quantified using feature Counts version 1.6.1, and count tables were generated based on Ensembl hg19 gene annotation gtf files. Differential expression analysis was conducted using the Bioconductor package DESeq2 version 1.12.4. All RNA-Seq data were deposited in the GEO database (Accession number: GSE 145938).

### CRISPR/dCas9-KRAB interference

For CRISPR interference, individual guide RNAs targeting the SE region of *MAGI2*, *ST3GAL6* and *ADM* were cloned into the lent guide-blasticidin plasmid and infected into dCas9-KRAB stable-expressing HMCLs. The successful transcriptional interference was confirmed by qRT-PCR and western blotting. The CRISPR (Addgene, Plasmid #71236) and inducible expression of guide RNA with fluorescent GFP reporter (Addgene, Plasmid #70183) vectors were gifts from Dr Takaomi Sanda. The sgRNA target sequences were listed in Supplemental Table 8.

### RT-PCR

Total RNA was extracted using RNeasy mini kit (Qiagen), and 1 μg aliquots were used for cDNA synthesis using the qScript™ cDNA Synthesis Kit (Quanta Biosciences). The cDNA templates were subjected to PCR amplification on CFX96 qPCR System (Biorad). Expression of each gene was normalized to GAPDH, and quantified using 2−delta(ct) method. Primers are listed in Supplementary Table 9.

### Gene ontology (GO) and signaling pathway analysis

Genes downregulated upon THZ1 inhibition were identified for further analysis. Gene ontology (GO) and Kyoto Encyclopedia of Genes and Genomes (KEGG) pathway enrichment analyses were conducted using DAVID software (https://david.ncifcrf.gov/chartReport.jsp).

### Statistical analyses

Nonparametric statistics were used with Prism 6.0 software (GraphPad Software, San Diego, CA, USA). Survival data were analyzed using the Kaplan–Meier method. Chi-square test was performed to analyze the categorical correlation. Student’s *t*-test and Mann–Whitney test were used to analyze parametric and nonparametric variables, respectively. Pearson correlation analysis was used to evaluate microarray data from a set of myeloma datasets. A *p*-value less than 0.05 was considered statistically significant.

## Discussion

SEs have been reported to be frequently associated with genes that define cell identity during normal development, and also be enriched at cancer gene loci in a range of malignancies^6,7^. In this study, we found SEs driving known oncogenes (*ST3GAL6* and *ADM)* as w critical subtype-specific drivers (*MAF* and *NUAK1*) in myeloma pathogenesis. Importantly, through profiling aberrantly epigenetic landscape and transcriptional program in myeloma cells, *MAGI2* was identified as a novel SE-oncogene related to MM disease progression and poor survival. Blockage of the SE region in *MAGI2* gene inhibited gene expression and impaired MM cell growth. Moreover, *MAF* bound SE locus and transactivated *MAGI2* expression. In summary, our research may provide a framework for the identification of novel SE-associated oncogenes, which are clinically and biologically relevant in MM.

The acquisition of SEs around oncogene drivers is widely observed in malignancies^29,30^_._ Here, we identified myeloma specific SEs that were absent in normal plasma cells and non-MM blood cancer cells, suggesting that they are acquired during the process of carcinogenesis. From our research and previous studies^8^, various critical oncogenes such as *MYC* and *IRF4* were all driven by its own SE, which may explain the aberrant high expression and dysfunction of these genes during carcinogenesis. Besides, we first reported that *NUAK1* was a SE-driven gene in t(14;16)-positive myeloma cell. Several potential *MAF* binding sites within the *NUAK1-*SE were found via online SE analysis tool^28^ and motif discovery with HOCOMOCO (data not shown here). The formation of SE with *MAF*-mediated transactivation at the *NUAK1* gene loci here could explain the overexpressed *NUAK1* in MAF- and MAFB-positive MM subgroups. Overall, the generation of abnormal SEs around key oncogenes could be a common tumorigenic mechanism in myeloma cells.

Bound by a very high density of TFs and cofactors (MED1, CDK7, and BRD4), SEs are susceptible to transcriptional inhibition^5,8^. Here, combining analysis of THZ1-responsive transcripts, MM-SE genes and clinical-relevant markers, we identified one novel SE-controlled oncogene, *MAGI2*. Previously, *MAGI2* was reported to interact with core proteins of multiple pathways, such as transforming growth factor-β signalling and Wnt/β-catenin signaling^24^. The critical role of *MAGI2* gene has been implicated in prostate cancer and hepatocellular cancers^31–33^. However, little was known about the relevance of *MAGI2* in MM pathogenesis prior to this study. Here, *MAGI2* gene was identified as a functionally relevant oncogene in myeloma, and depression of the *MAGI2*-SE region markedly attenuated cell proliferation and induced cell apoptosis. We further demonstrated a novel mechanism of oncogene upregulation through oncogenic TF, in this case the transcription factor *MAF*, which *MAF* itself is upregulated as a result of its juxtaposition to IgH enhancer (MAF-translocated t(14;16)) or de novo SE formation, binding to SE formed in the oncogenic process. Our findings suggested *MAGI2* is a functionally SE-controlled oncogene in development of MM, and the downstream targets and its related pathways are still under further investigation.

Normally, SEs function through cooperative and synergistic interactions with multiple TFs and coactivators. In the case of *MAGI2*, we identified the transcription factor—*MAF*—that bound the *MAGI2-*SE element and activated expression of the SE-related oncogene. The oncogenic transcription factor *MAF* is translocated in ~5–10% of MM patients, which is associated with the malignant process and *MAF* overexpression confers a poor prognosis^26,27^. The frequent overexpression of *MAF* in MM makes it an attractive therapeutic target. Here, despite the observed positive correlation of *MAF* and *MAGI2* expression in HMCL and MM datasets, we also confirmed significant downregulation of *MAGI2* upon shRNA-mediated knockdown of *MAF* in two *MAF*-translocated HMCL, MM1.S, and JJN3. Active histone modifications H3K27ac as well as transcriptional coactivators (BRD4 and MED1) associated with SEs element enriched at *MAF* binding sequences were characterized in *MAF* overexpressed MMCL. Together, SE-driven *MAGI2* gene is an essential driver of oncogenic phenotype in MM and are further accentuated by oncogenic *MAF* interactions. This *MAF*/SE/*MAGI2* regulatory pathway may be a unique oncogenic mechanism that is amenable to MM therapeutic intervention.

In conclusion, we demonstrated that SE-associated genes, especially those that are transcriptionally dependent, are often of clinical and biological relevance to myeloma, and some of these dependencies could be exploited for potential therapeutic targets. In addition, transcription activation of SE-associated genes by oncogenic TF activated by specific genetic abnormalities could be a novel mechanism for genetic subtype-specific oncogenic activation.

## Conclusion

Functional genes marked by SE are related to cell fate‐determined and pathological processes of MM, and these data suggests a strategy for further human MM clinical investigation. Importantly, the *MAF*/SE/*MAGI2* regulatory circuit could potentially represent an attractive therapeutic target for future regenerative and cell-based myeloma therapies.

## Supplementary information

Supplementary methods and materials

Supplementary Figure-1

Supplementary Figure-2

Supplementary Figure-3

Supplementary Figure-4

Supplementary Figure-5

Supplementary Figure-6

Supplementary Table 1

Supplementary Table 2

Supplementary Table 3

Supplementary Table 4

Supplementary Table 5

Supplementary Table 6

Supplementary Table 7

Supplementary Table 8

Supplementary Table 9
