# Supplementary material for: Myeloma-specific superenhancers affect genes of biological and clinical relevance in myeloma
Source: Blood Cancer J. 2021 Feb 12;11(2):32. doi: 10.1038/s41408-021-00421-7 (PMC7881003; doi:10.1038/s41408-021-00421-7)
Supplement: Supplementary file 1 — Supplementary methods and materials [file 41408_2021_421_MOESM1_ESM.docx]

**Supplementary methods and materials**

## **Immunoblotting assay**

Cells were lysed in RIPA lysis and extraction buffer (Thermo Fisher Scientific), supplemented with proteinase inhibitor cocktail and phosphatase inhibitor cocktail (Roche) for 30 min on ice. Protein quantification was determined by Bradford assay. Immunoblotting was performed using SDS-PAGE followed by protein transfer to PVDF membrane (Bio-Rad). Primary antibodies were incubated overnight in cold room. Secondary antibodies were incubated for 1 h at room temperature. The following antibodies were used: GAPDH: (1: 500, SANTA CRUZ BIOTECHNOLOGY, sc-47724); β-Actin (1:1000, Rabbit mAb#4970 Cell Signaling Technology); RNA polymerase II CTD repeat YSPTSPS (phospho S2)(1:1000, Abcam, ab5095), RNA polymerase II CTD repeat YSPTSPS (phospho S5) (1:1000, Abcam, ab5153), RNA polymerase II CTD repeat YSPTSPS (1:1000, Abcam, ab817); CDK7 (1:500, Cell Signaling Technology, #2090); PARP（1:1000, Cell Signaling Technology, #9542）; CAS9 (1:1000, Cell Signaling Technology, #14697); MAF (1:1000, SANTA CRUZ BIOTECHNOLOGY, sc-518062); MAGI2 (1:1000, Thermo Fisher Scientific, PA5-21601). The signals were detected with SuperSignal reagents (Thermo Fisher Scientific) on an ImageQuant LAS500 (GEHealthcare).

## **shRNA knockdown and transfection.**

MM cell lines, KMS11 and JJN3, were transfected with short hairpin (shRNA) plasmid using Neon Transfection system (Life Technology) according to manufacturer’s protocol (KMS11: 1050V, 30 ms, 1 pulse; JJN3: 1150V, 20 ms, 3 pulse). pLko.1 scrambled shRNA was purchased from Sigma-Aldrich. The four-independent *MAF* shRNA were purchased from Sigma-Aldrich Mission TRC shRNA library. Cells were harvested at 48H for protein extraction. *MAF* shRNA Target Region and shRNA Sequence were listed below.

| shRNA | Target Region | shRNA Sequence |
| --- | --- | --- |
| 1 | CDS | CCGGCAAGGAGAAATACGAGAAGTTCTCGAGAACTTCTCGTATTTCTCCTTGTTTTT |
| 2 | CDS | CCGGTGGAAGACTACTACTGGATGACTCGAGTCATCCAGTAGTAGTCTTCCATTTTT |
| 3 | CDS | CCGGTGGAAGACTACTACTGGATGACTCGAGTCATCCAGTAGTAGTCTTCCATTTTTG |
| 4 | 3UTR | CCGGTTTATGGTGTGTGCAAGTAAACTCGAGTTTACTTGCACACACCATAAATTTTTG |

## **Flow cytometry for cell-cycle and cell apoptosis analysis**

Cell apoptosis was measured using flow cytometric analysis of double staining with Annexin V and propidium iodide (PI) according to the manufacturer’s instructions (BD Biosciences). Data were analyzed using FlowJo 7.6 software (Tree Star). For cell-cycle analysis, cells were harvested and washed twice with PBS, and the Cell Cycle Staining Kit (MULTISCIENCES, CCS012) was used. Cell apoptosis and cell-cycle analyses were performed by flow cytometry (Accuri model C6).

## **Chromatin immunoprecipitation (ChIP) assay**

ChIP assays were performed using reagents obtained from Cell Signaling Technology (SimpleChIP® Enzymatic Chromatin IP Kit (Magnetic Beads) #9003). Chromatin was used for immunoprecipitation with anti-*MAF* and normal rabbit-IgG antibody as negative control. ChIP-enriched DNA was measured using real-time PCR, and the primer sets for the *MAGI2* SE were designed as follows:

| Primer | Forward | Reverse |
| --- | --- | --- |
| E1 | 5’-GGTGAGGCGTGAATTCCTCT-3’ | 5’-TTGAGACGCCCATGAGACAC-3’ |
| E2 | 5’-GCTGAGAAAGGCCAAGCAAT-3’ | 5’-GGAGAGGAATTCACGCCTCA-3’ |
| E3 | 5’-CACTGGCAAGGGGATTGGTG-3’ | 5’-CTCAGGAGCAGTGTGAGAGTG-3’ |
| E4 | 5’-CAAACCCTGTTCCTACACCAGT-3’ | 5’-CCCTTGCCAGTGTGTGAAGT-3’ |

## **Sequencing data legends**

ChIP-seq was performed on MM patients-derived samples and cell lines using polyclonal anti-H3K27Ac. RNA-Seq was performed in JJN3 and H929 cells treated with THZ1 (50nM, 24hr) or with the vehicle control. The complete raw data of H3K27ac ChIP-Seq and RNA-Seq were deposited in NCBI's Gene Expression Omnibusis (GEO) and are accessible at <https://www.ncbi.nlm.nih.gov/geo/query/acc.cgi?acc=GSE145938>.

## **Supplementary Table legends**

**Supplementary Table 1:** Clinical and genetic information on ten patients-derived MM cases, with features of common clinical factors, genetics and cytogenetics, translocations involving chromosome IgH region, and clinical outcomes.

**Supplementary Table 2:** Translocations and cyclin D expression (TC)-based classification system of human myeloma cell lines included in this study.

**Supplementary Table 3:** Identified superenhancers and superenhancers-associated genes in lymphoma B cells, three memory B cells and normal plasma cell, with detailed information including super enhancer region, rank of enhancer signals and distance to target gene.

**Supplementary Table 4:** Identified super enhancers and super enhancers-associated genes in selected HMCLs (KMS12, KMS11, H929, U266, RPMI-8226, KMS28BM and JJN3), with detailed information including super enhancer region, rank of enhancer signals and distance to target gene.

**Supplementary Table 5:** Identified super enhancers and super enhancers-associated genes in patients-derived MM tissues (MM 1-10), with detailed information including super enhancer region, rank of enhancer signals and distance to target gene.

**Supplementary Table 6:** Clinical significance of selected MM-SE genes in Mayo and Italy myeloma database. A p-value less than 0.05 is statistically significant.

**Supplementary Table 7:** A web tool SE analysis was used to perform an enrichment analysis to identify super-enhancer associated transcription factor (TF) binding sites across the *MAGI2*-super enhancer region (http://licpathway.net/SEanalysis).

**Supplementary Table 8:** Designed gRNA sequence that recognized the target DNA region of *MAGI2, ADM, ST3GAL6* super enhancer and directed the Cas nuclease for genome editing.

**Supplementary Table 9:** RT-qPCR primers are designed to amplify selected MM-SE genes.

## **Supplementary Figure legends**

**Supplementary Fig. 1.**

**A.** Enhancer regions in primary MM and MM cell lines were ranked by increasing H3K27ac signal, and enhancers above the inflection point of the curve were defined as SEs. The total number of SEs was shown for each sample.

**Supplementary Fig. 2.**

**A, B.** Normalized mRNA expression of *ST3GAL6* (A) and *ADM* (B) in BMPC and various hematological malignancies. **C.** Based on Mayo myeloma dataset, mRNA expression levels of *ST3GAL6* (left) and *ADM* (right) were correlated with MM progression and stages. **D.** High expression of *ST3GAL6* and *ADM* predicted unfavorable MM patient’s survival**. E, F**. The ChIP-seq gene tracks represent H3K27ac signal in NPC, MBC, B-lymphoma cells, MM cell lines and primary MM samples at the *MAF* (E) and *NUAK1* (F) gene loci with or without the indicated cytogenetic abnormality. BMPC: Bone marrow plasma cell; AML: Acute myeloid leukemia; ALL: Acute lymphocytic leukemia; CLL: Chronic lymphocytic leukemia; CML: Chronic myelogenous leukemia; DCBCL: Diffuse large B-cell lymphoma.

**Supplementary Fig. 3**

**A.** A panel of MM cell lines were tested for response to THZ1 (5–1000 nM, 48 hr) by measurement of ATP levels (left). The IC50 values of THZ1 against MM cell lines were presented (right). **B.** JJN3, H929 and KMS11 cells were treated with indicated THZ1 for 48 hours, and cell apoptosis was measured by FACS analysis. Treatment of THZ1 induced JJN3 and H929 cell apoptosis, but exerted limited effect on KMS11 cells. **C.** Expression levels of TE-associated genes (GAPDH and TUBA1A) upon indicated THZ1 treatment for 24 hours.

**Supplementary Fig. 4**

**A, B**. The mRNA expression levels of selected MM-SE genes in JJN3 (A) and KMS11 (B) cells upon THZ1 treatment (50 nM, 24 h) with vehicle as control. **C**. The mRNA expression levels of selected MM-SE genes in JJN3 cells upon JQ1 treatment (50 nM, 24 h) with vehicle as control.

**Supplementary Fig. 5**

**A.** Chromatin occupancy of H3K27ac on *MAGI2*-SE region by target-specific (sgRNA_1, sgRNA_2) or nontargeting sgRNA in JJN3 cell. ChIP signals (Fold Enrichment) are shown as mean ± SEM. *p<0.05, **p<0.01, ***p<0.001, ****p<0.0001 by two-sample, two-tailed t-test compared with the controls. Experiments were performed in triplicate. **B, C.** Deletion of *MAGI2-*SE in MM1.S cells caused promoted cell apoptosis (B) and an increased proportion of sub-G1 cells (C).

**Supplementary Fig. 6**

**A**. Normalized mRNA expression level of *MAF* in different genetic subtypes of MM (data based on CoMMpass, UAMS and Mayo myeloma datasets). **B**. The expression level of *MAGI2* is the highest in the *MAF*-positive subgroup across various independent MM datasets (GSE4581 and GSE9782). **C**. Pearson's correlation coefficient was used to measure the statistical relationship between *MAGI2* and *MAF* expression in a set of myeloma datasets. High positive correlation were seen for the *MAGI2* and *MAF* genes.
