# Supplementary figures and images for: Myeloma-specific superenhancers affect genes of biological and clinical relevance in myeloma
Source: Blood Cancer J. 2021 Feb 12;11(2):32. doi: 10.1038/s41408-021-00421-7 (PMC7881003; doi:10.1038/s41408-021-00421-7)

Supplementary Figure-1

A

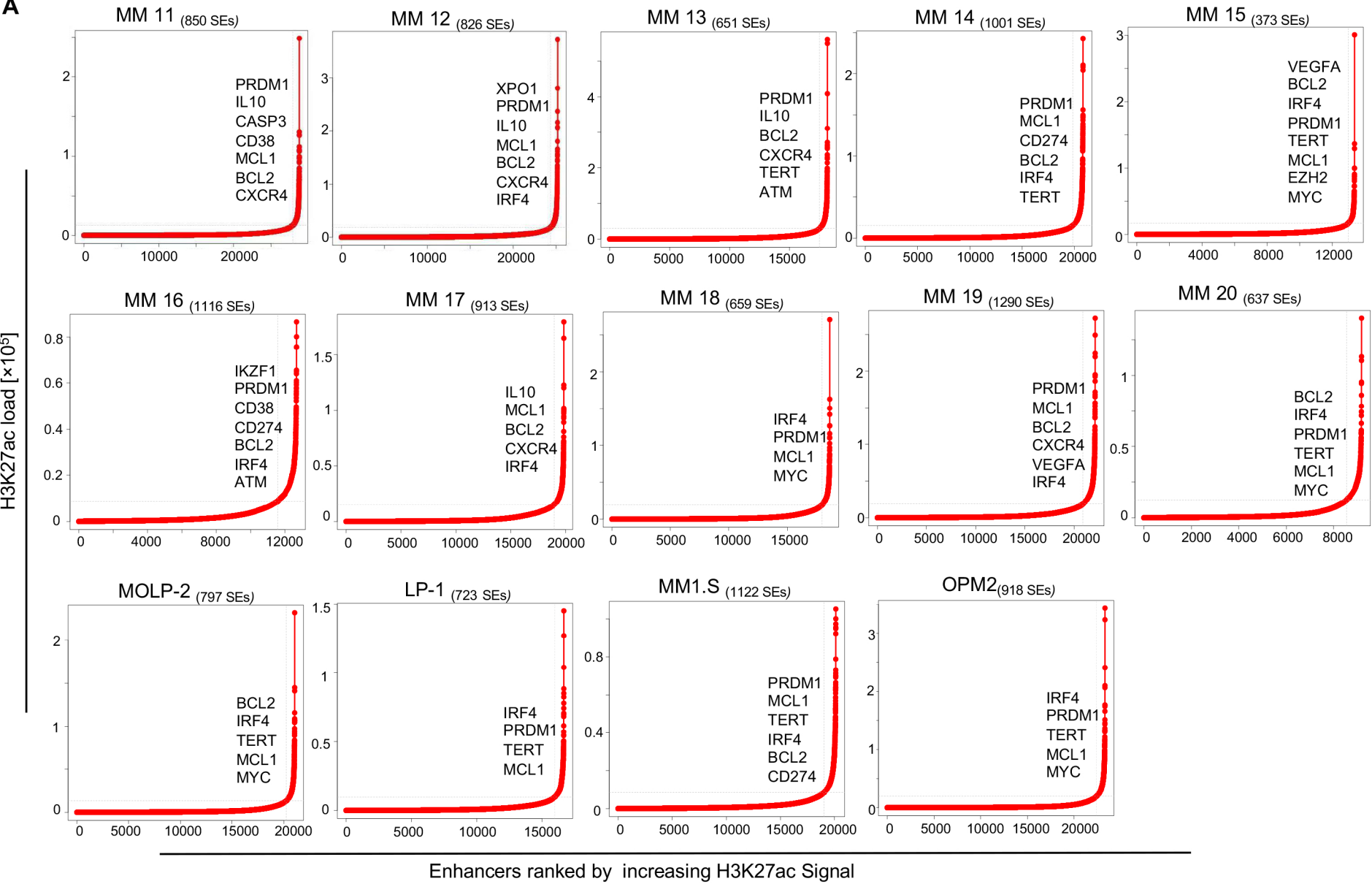

Supplement: Supplementary file 2 — Supplementary Figure-1 [file 41408_2021_421_MOESM2_ESM.pdf]

Supplementary Figure-2

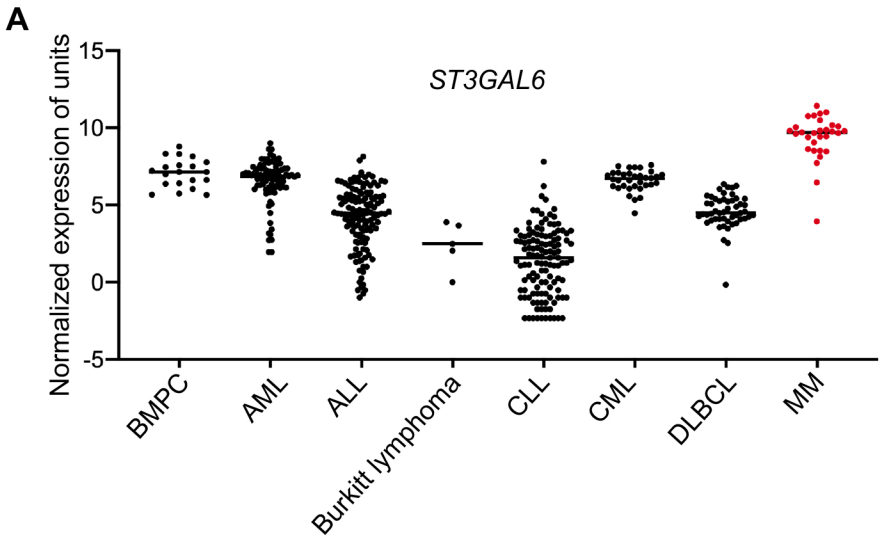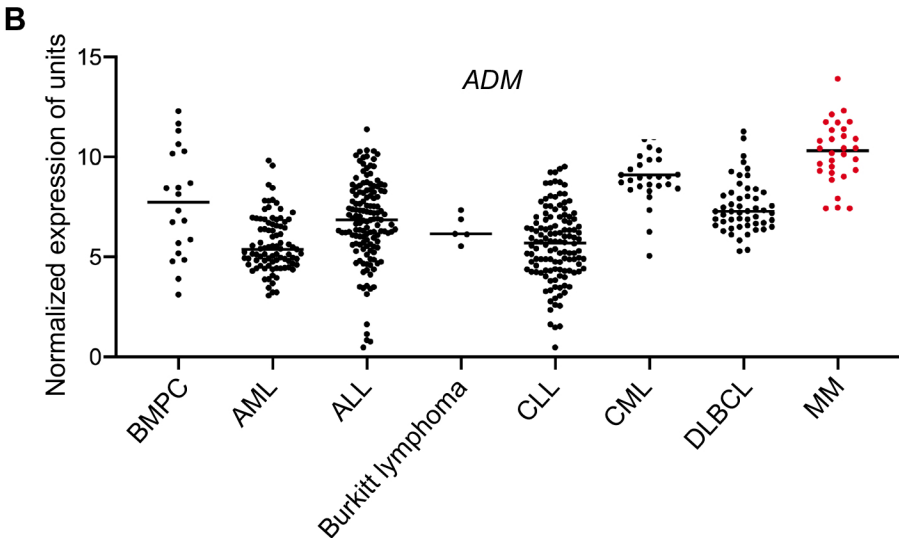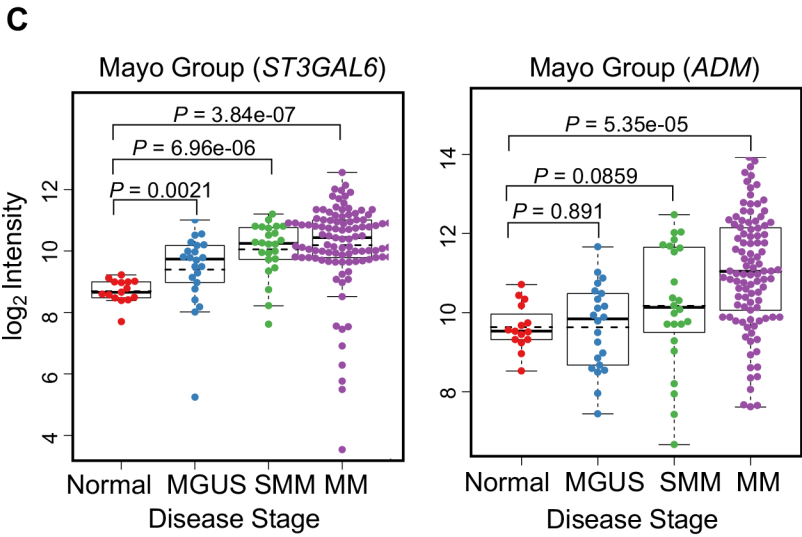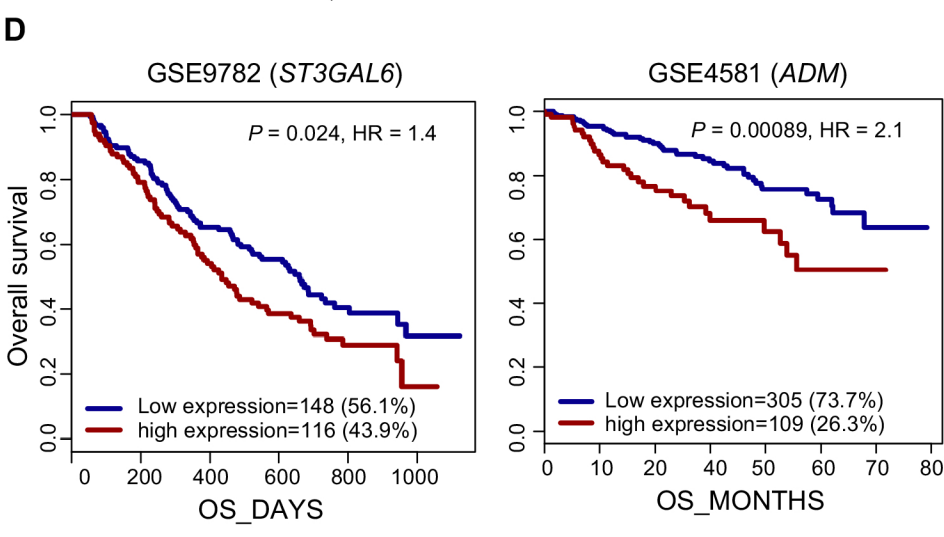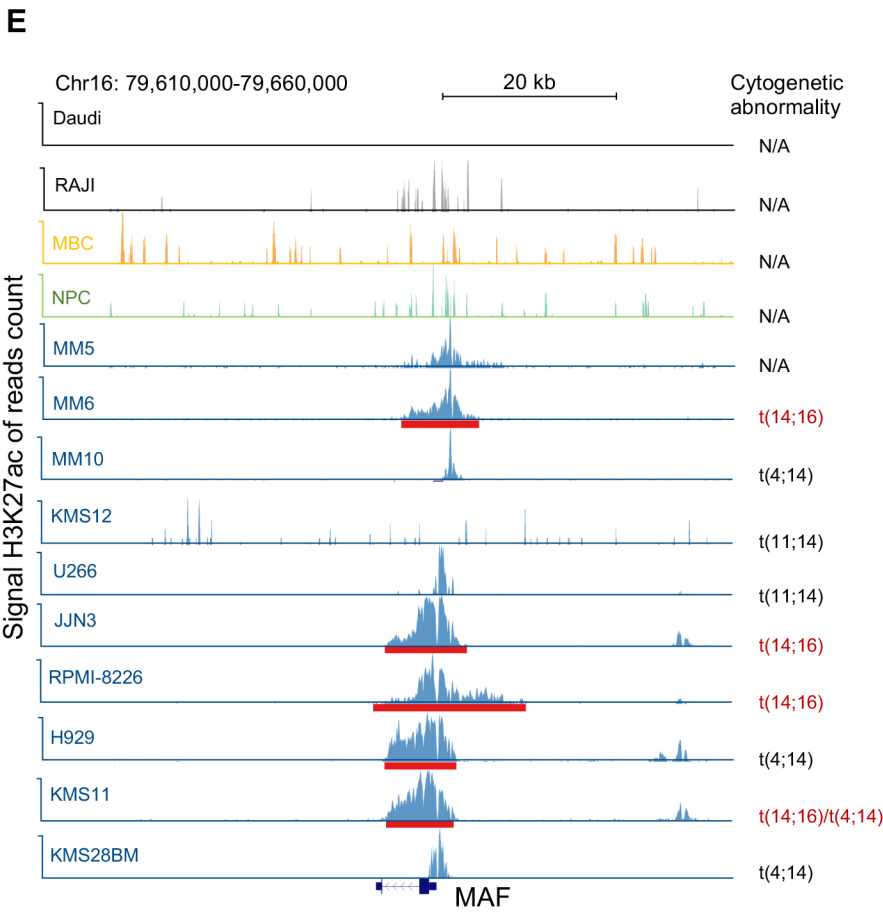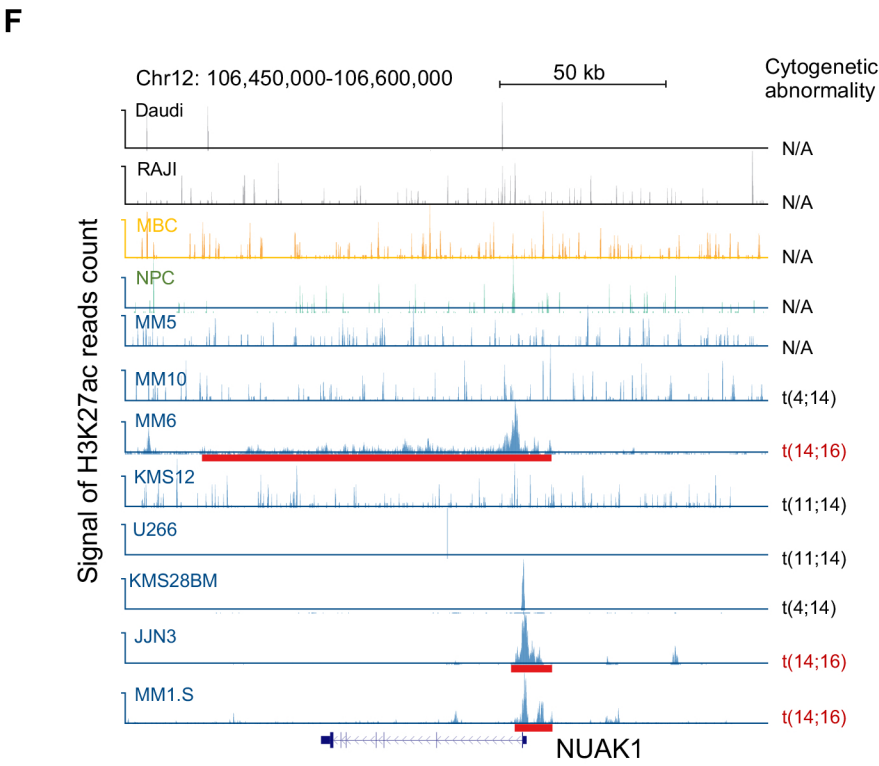

Supplement: Supplementary file 3 — Supplementary Figure-2 [file 41408_2021_421_MOESM3_ESM.pdf]

Supplementary Figure-3

A

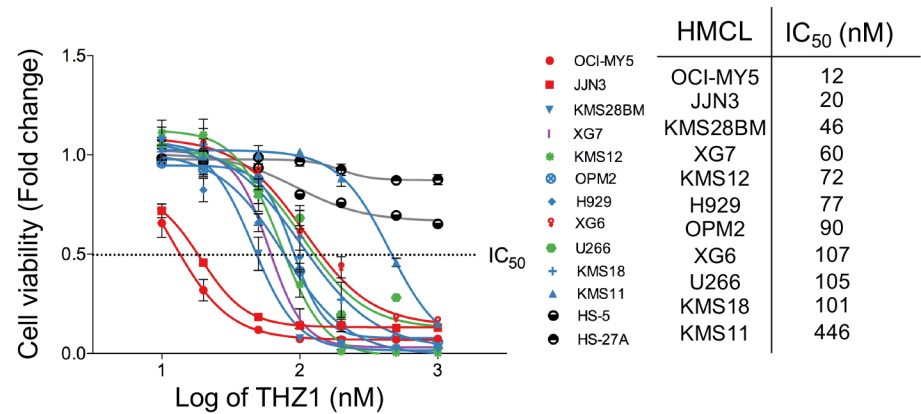

B

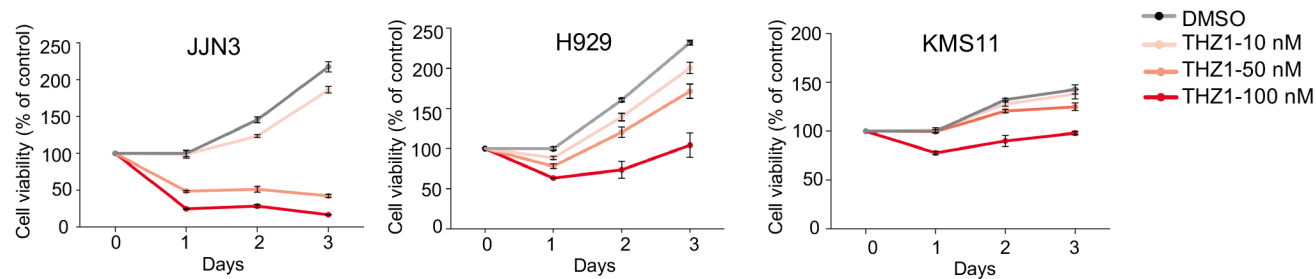

C

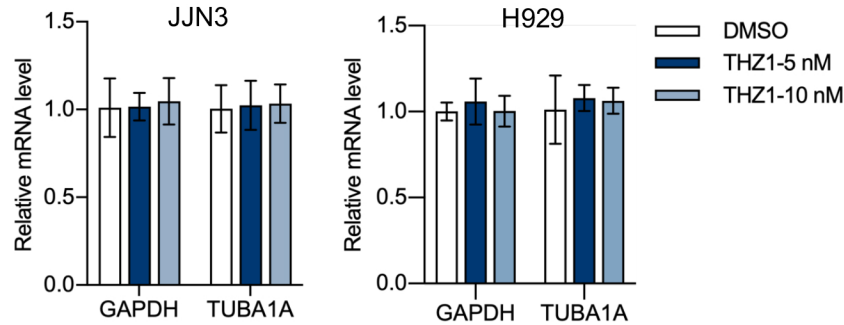

Supplement: Supplementary file 4 — Supplementary Figure-3 [file 41408_2021_421_MOESM4_ESM.pdf]

Supplementary Figure-4

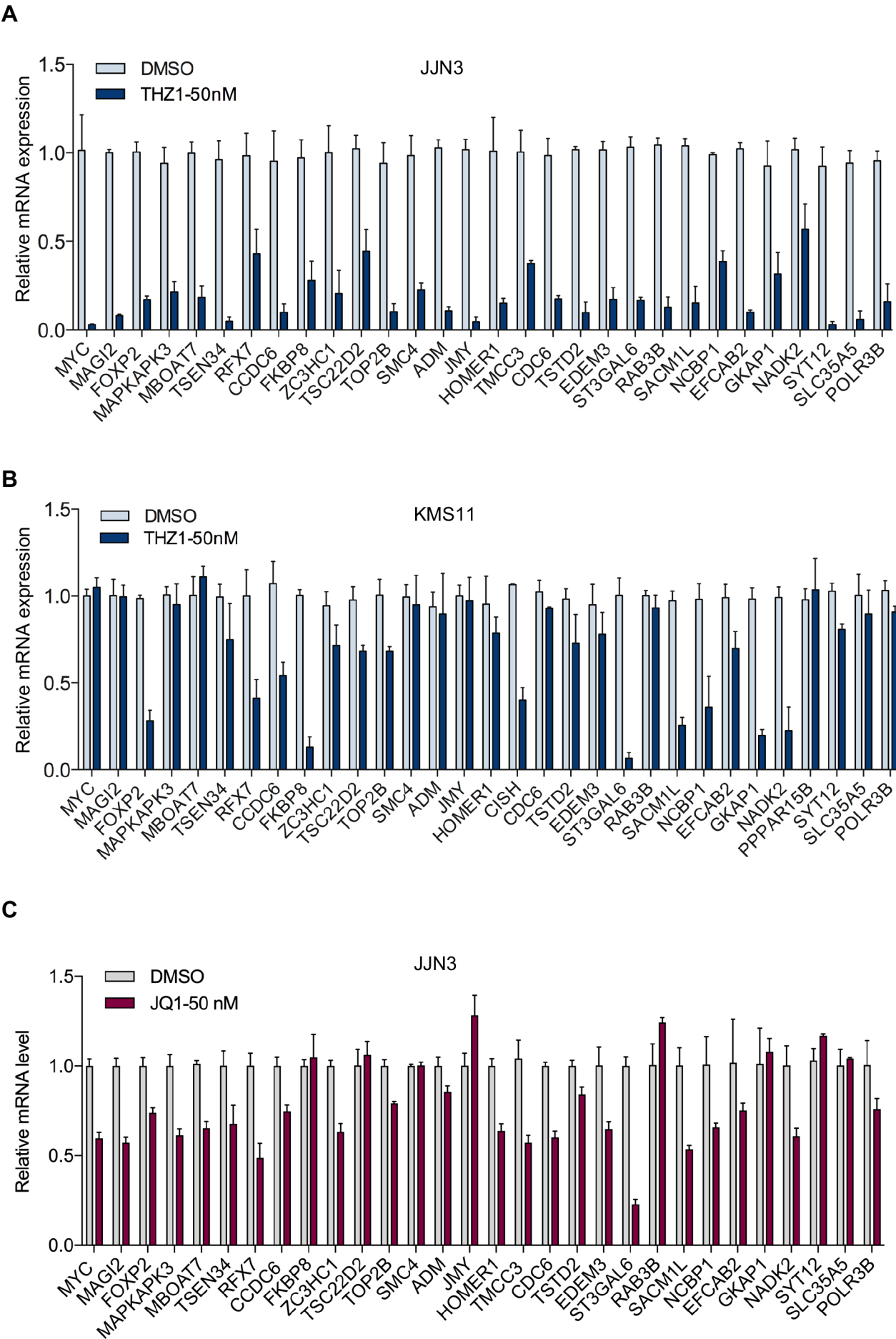

Supplement: Supplementary file 5 — Supplementary Figure-4 [file 41408_2021_421_MOESM5_ESM.pdf]

Supplementary Figure-5

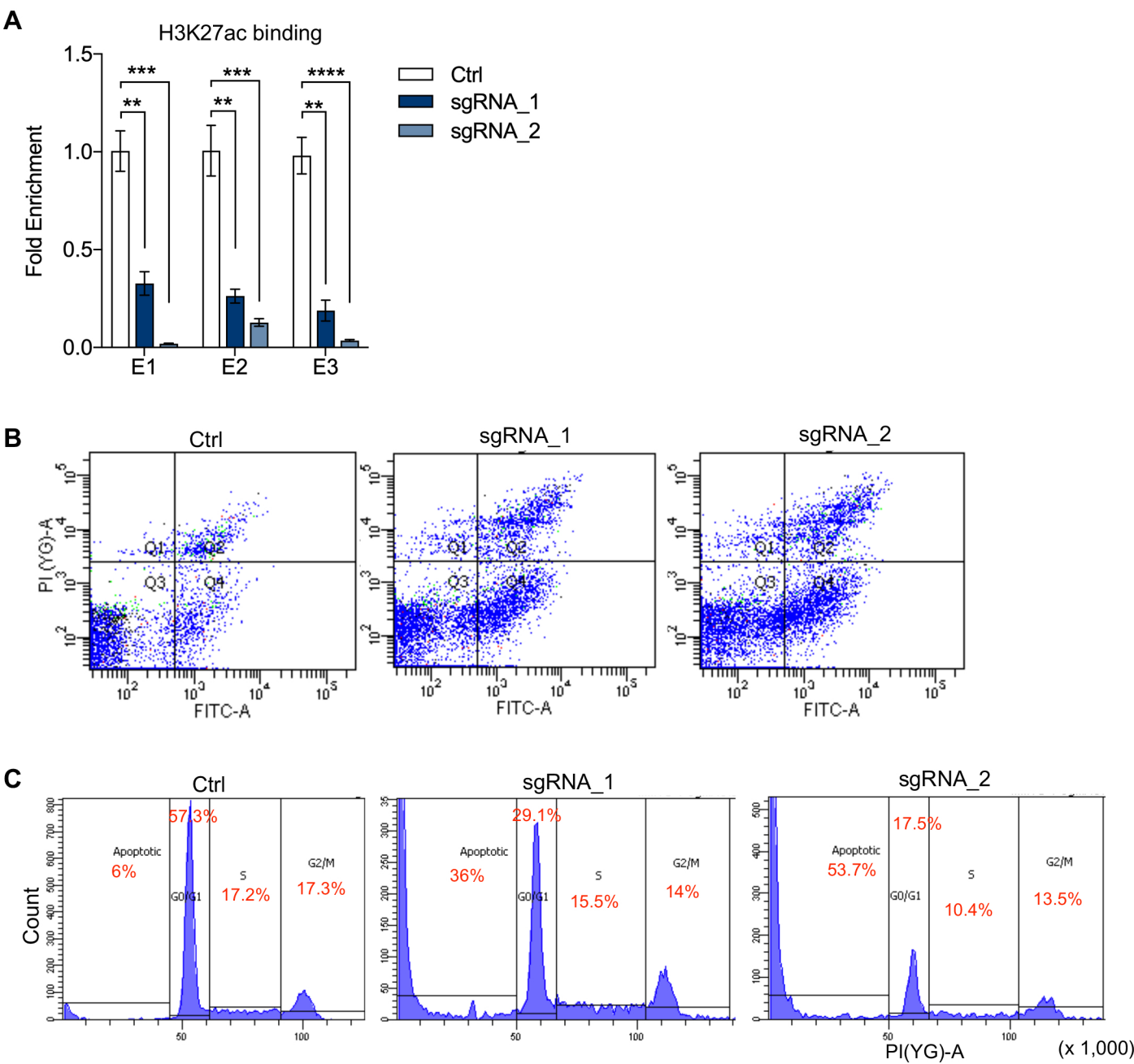

Supplement: Supplementary file 6 — Supplementary Figure-5 [file 41408_2021_421_MOESM6_ESM.pdf]

Supplementary Figure-6

A

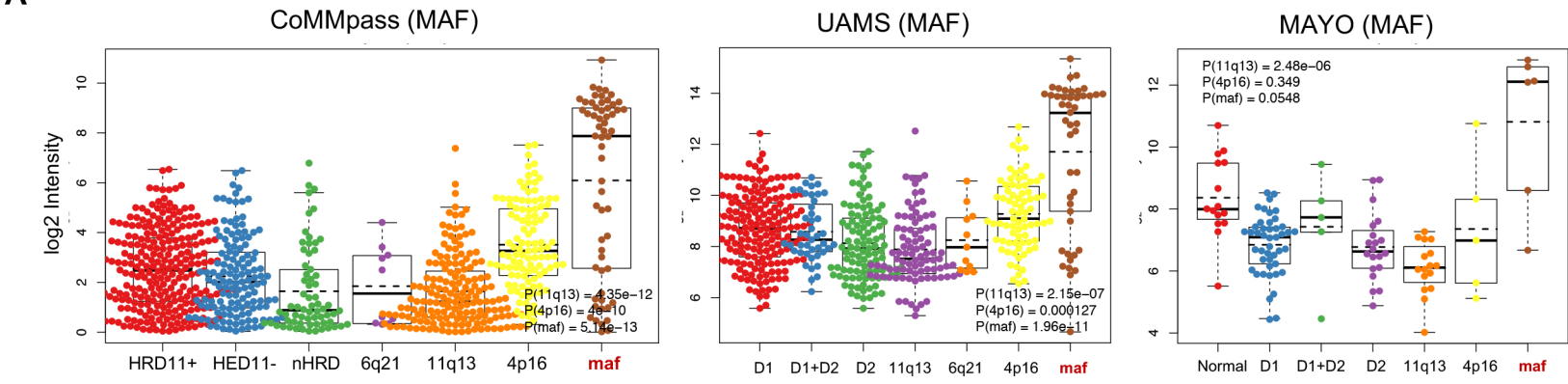

B

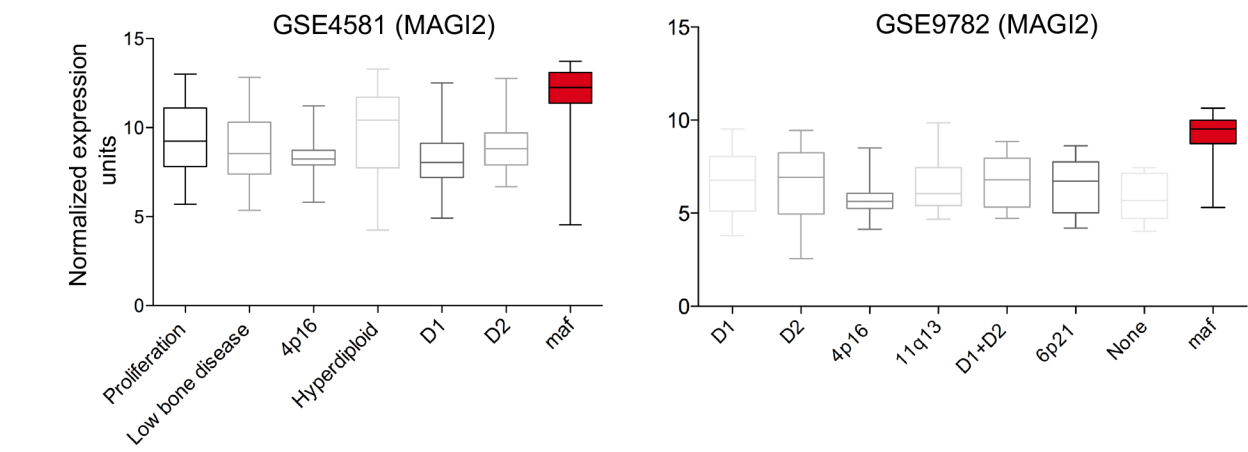

C

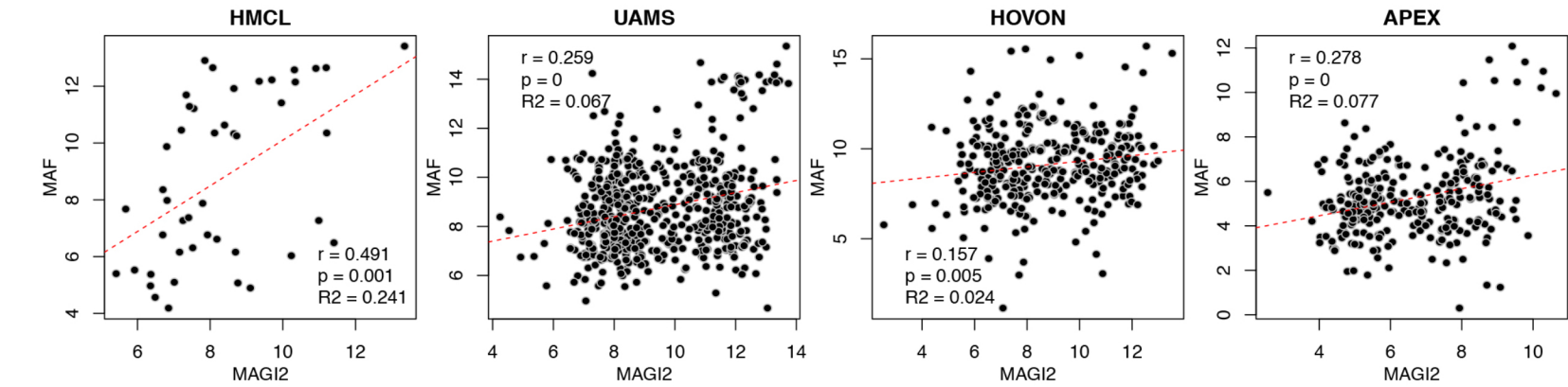

Supplement: Supplementary file 7 — Supplementary Figure-6 [file 41408_2021_421_MOESM7_ESM.pdf]
